# Image Translation by Domain-Adversarial Training

**DOI:** 10.1155/2018/8974638

**Published:** 2018-06-26

**Authors:** Zhuorong Li, Wanliang Wang, Yanwei Zhao

**Affiliations:** College of Computer Science and Technology, Zhejiang University of Technology, Hangzhou 310023, China

## Abstract

Image translation, where the input image is mapped to its synthetic counterpart, is attractive in terms of wide applications in fields of computer graphics and computer vision. Despite significant progress on this problem, largely due to a surge of interest in conditional generative adversarial networks (cGANs), most of the cGAN-based approaches require supervised data, which are rarely available and expensive to provide. Instead we elaborate a common framework that is also applicable to the unsupervised cases, learning the image prior by conditioning the discriminator on unaligned targets to reduce the mapping space and improve the generation quality. Besides, domain-adversarial training inspired by domain adaptation is proposed to capture discriminative and expressive features, for the purpose of improving fidelity. Effectiveness of our method is demonstrated by compelling experimental results of our method and comparisons with several baselines. As for the generality, it could be analyzed from two perspectives: adaptation to both supervised and unsupervised setting and the diversity of tasks.

## 1. Introduction

While humans can easily translate an image into another image, for instance, imagining the missing region of corrupted image or sketching the scenic photograph, it is challenging for machine to automatically learn the mapping [1, 2], especially when supervision is absent. Similar tasks include image colorization [3, 4], image inpainting [5], image semantic segmentation [6], and image denoising [7]. All of these can be framed as image-to-image translation [8] as they share the fact that they could be formulated as pixel regression or classification [9]. For instance, depth and surface normal estimation in [10, 11] were tasks that translate a color image into a geometric output image by optimizing a pixel regression formulation, while [12] translated the given photos into semantic labels by tackling a pixel classification problem. However, these methods are all pixel-wise matching oriented, being inadequate for high-level representations and tend to yield blurry outputs [13]. In parallel, recent studies had shown that feature-wise loss, which is based on discrepancy in hierarchical representation of deep neural networks, leads to sharper synthetic images in certain tasks like image style transfer [8, 14] and superresolution [15]. Nevertheless, using the feature-wise loss alone could not guarantee realism and tends to induce high-frequency artifacts.

The recently emerging Generative Adversarial Network (GAN) [16] that proposed as a generative model had attained empirical successes in image generation, being a promising alternative to the methods above for image translation [1, 13, 17]. Recent work [18] moved beyond specific image translation tasks by developing a GANs-based common framework for various image translation tasks. Such method requires explicitly aligned data in the form of* (input image*,* ground-true)* for training, which is seldom available in practice. For instance, on the gender swapping in image editing, no data pair as* (male*,* female)* is available concerning the same person. Attempts to alleviate this issue had also been made to exploit dual learning [2, 19, 20] with cyclic loss [21]. However, cycle-consistency enforced via *ℓ*_1_ or *ℓ*_2_ loss tends to induce the averaging of potential location of details and thus leads to oversmoothed images.

**Figure 1:**
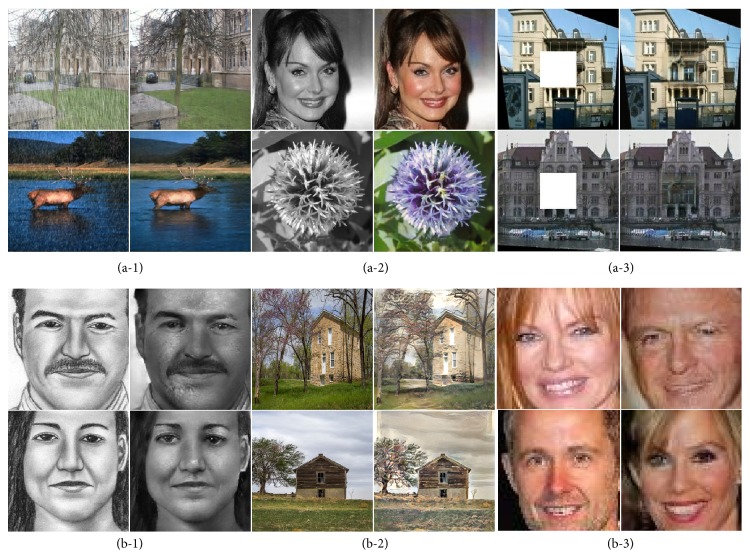
Our approach learns to “translate” an input image into the one of desired character with or without aligned data: (a-1) image deraining; (a-2) image colorization; (a-3) image inpainting; (b-1) sketch to photo; (b-2) image style transfer; and (b-3) gender swapping.

In this paper we develop a conditional GAN-based framework that is adapted to both aligned and unaligned image translations, each of which would traditionally involve specific formulations with expert knowledge. Cross-domain relations are learned by cyclic loss as well as conditioning the discriminator on unaligned targets, which reduces the uncertainty of mapping from an individual input to the desired output. Besides, we propose a domain-adversarial training method that pushes the discriminator to additionally distinguish the reconstructed image, thus learning more expressive features of image generation. Extensive evaluations have been performed on various image translation tasks, showing that plausible results can be attained by the proposed method. Several examples of our translated images can been seen in Figure 1.

The remainder of this paper is organized as follows.  Section 2 gives brief introduction to related theory and previous studies on image translation.  Section 3 is dedicated to an explanation of the proposed method. In Section 4, implementation is described in detail.  Section 5 presents experiments for the evaluation of the proposed approach. Finally, conclusions and perspectives for future works are presented in Section 6.

## 2. Related Work

### 2.1. Conditional Generative Adversarial Networks

GANs have been proposed as a novel approach to train a generative model, which contain a pair of parametric networks. A discriminative network is trained to distinguish the generated samples from the real ones, while the generative network intends to produce realistic samples to fool the discriminator. GANs are just right for diverse image-to-image translation since only images that are plausible enough would be accepted by the trained discriminator despite specific applications. Impressive success can be found in image generation [22, 23] and image editing [24–27]. Extensive works have been proposed to improve the training or to explore the applications. An important line of works, which are referred to as conditional generative adversarial networks (cGANs) [28], attempt to address the unstable training by introducing a conditional variable to both networks. Methods included in this line are applied to image2image [29], text2image [30], texture synthesis [31], image inpainting [1], and frame prediction [32]. Despite difference in the form of applications, they share a common key to success, that is, producing desirable images by introducing a prior.

### 2.2. Domain Adaptation

Domain adaptation is proposed to learn representations that are invariant to data from different distributions [33]. In other words, cross-domain relation in the form of a mapping from source domain to target domain needs to be built, which is quite similar to our goal in image-to-image translation. General approaches to domain adaptation focus on the representations that are linear [34, 35] or nonlinear [36, 37]. Alternatives that have been recently proposed can be roughly categorized into two classes: the first class is based on unsupervised learning while the other is supervised. For the former, feature space distribution is matched by means of samples reweighting [38, 39] or geometric transformation in feature space [33, 40, 41]. When it comes to the supervised case, approaches focus on how to exploit the labeled data form the target domain [42, 43].

### 2.3. Deep Image Representation

Utilizing the activation discrepancy of classifier's intermediate layers between output and target tends to yield sharper visual results, as it better represents complex features and complements the ordinary pixel-wise disparity [44, 45]. The feature discrepancy could be defined by the combination of activation layers of pretrained deep neural networks such as VGG19 [46] or a part of the discriminator or the generator network [13]. The feature discrepancy provides considerable gradients for generator *G* to be optimized to encourage the perceptual similarity between the translated image and the reality, rather than just forcing them to be exactly the same in pixel values.

Our work is relevant to [18] with respect to the cGAN-based common framework for diverse image translation tasks, as well as [19, 20] in terms of the cross-domain relation learning. However, on the one hand, generators in [18, 20] are trained with a pixel-wise loss, which imposes a hurdle for expressive representation learning. By contrast, we simultaneously minimize the feature discrepancy and pixel-wise loss. On the other hand, the discriminator in [19] is unconditional and thus unexpectedly increases the underdetermination. To address this problem we condition the discriminator on the unaligned target as an image prior. In addition, our method implements the idea of domain adaptation in the context of GAN to capture deep image features and further sharpen the translated images.

## 3. Methods

**Figure 2:**
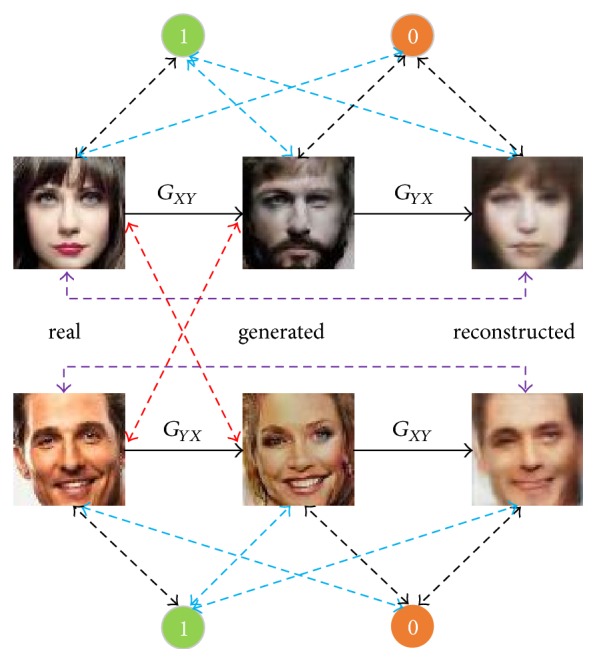
Scheme of our model. Solid lines denote the forward data flow while dash lines with two-headed arrow denote the losses. Specifically, black dash lines and blue dash lines are adversarial losses of the discriminator and generator separately. Pixel-wise and feature-wise losses are, respectively, denoted with the violet dash lines and red dash lines.

Our goal is to learn two mappings *G*_*XY*_ and *G*_*YX*_ given training set {*x*_*i*_}_*i*=1_^*m*^ ∈ *X* and  {*y*_*j*_}_*j*=1_^*n*^ ∈ *Y*. When *m* ≠ *n*, this is referred to unaligned image translation. As illustrated in Figure 2, *G*_*XY*_ maps images from domain *X* to *Y* and *G*_*YX*_ does in an opposite way. The images that the *G*_*YX*_ generates are constrained to be the reconstructions of images in domain *X*. Discriminator *D*_*Y*_ is introduced to distinguish images among generated images {*G*_*XY*_(*x*)} (labeled with 0), reconstructed images {*G*_*YX*_(*G*_*XY*_(*x*))} (labeled with 0), and the inputs {*x*} (labeled with 1). *D*_*X*_ works in the same way as *D*_*Y*_ does. Note that unaligned images in the cross-domain are conditional variables for the discriminator. In addition to domain-adversarial training with the generator, discriminator in our architecture also acts as a feature comparator to match the latent representations of the translated image and those of the images in the target domain. Our full objective consists of three components: a domain-adversarial loss that utilizes the unaligned data in the target domain as the conditional information to encourage the realism of the transformed images as well as to capture the data distribution; a deep features discrepancy between translated image and the unaligned target of input to learn the most discriminative characteristics of the target domain; a pixel-wise error to guide the appearance of the outputs. Each loss component is elaborated in the following subsections.

### 3.1. Domain-Adversarial Loss

GANs [16, 28] simultaneously train a parametric discriminator network *D* to classify whether the image received is the ground truth or is produced by the generator network *G*, and *G* to confuse *D* by yielding outputs with realistic appearance. GANs can serve as a common solution for diverse image-to-image mappings since it can learn a loss adapted to the data. Formally, the adversarial loss can be written as

(1) minG⁡maxD⁡ LGAN=Elog⁡Dx+Elog⁡1−DGz,

where *x* is the observed image and *z* is the random noise vector mapped to the output image by the generator *G*.

To better capture the underlying distribution of the target domain *Y*, we condition the discriminator on the target images. To be more specific, the condition variable is the unaligned target *y*′, which is the real image in target domain *Y* but is not paired with the input *x*, drawing the output images to the manifold of the target images. The unaligned target *x*′ in domain *X* works in the same way as *y*′. Besides, previous works [1, 18] have shown that improved results can be achieved when the generator is conditioned without the noise variables *p*_*z*_(*z*) and further replaced by a transformation network akin to an autoencoder. Formally, the unaligned conditional adversarial loss we propose can be formulated as

(2) LDYcGAN=−Elog⁡Dy,y′−Elog⁡1−DGXYx,y′,

(3) LDXcGAN=−Elog⁡Dx,x′−Elog⁡1−DGYXy,x′,

(4) LGXYcGAN=−Elog⁡DGXYx,y′,

(5) LGYXcGAN=−Elog⁡DGYXy,x′.

Note that the unaligned target *y*′ is replaced with the ground-true *y* and the cyclic loss is not necessary in the supervised case:

(6) LaligncGAN=minGmaxD⁡ Elog⁡Dy,y+Elog⁡1−DGx,y.

The discriminator can easily detect the discontinuity and unnatural appearance of the generated sample and then reject it with high confidence. This is especially the case in the early stage of training. Consequently the generator could not get sufficient gradient to learn well. To address this issue, we elaborate a way to make the discriminating process tougher. We modify the plain adversarial loss and force the discriminator to further detect the reconstructed image besides the binary labeling that is real or fake. From the viewpoint of domain adaptation, the discriminator of our proposed is a classifier that is trained to learn the cross-domain relations among real data, generated data, and the reconstruction. Domain-adversarial loss can be formulated as

(7) LdaDY=−Elog⁡Dy,y′−Elog⁡1−DGXYx,y′−Elog⁡1−DGYXGXYx,y′,

(8) LdaDX=−Elog⁡Dx,x′−Elog⁡1−DGYXy,x′−Elog⁡1−DGXYGYXy,x′,

(9) LdaGXY=−Elog⁡DGXYx,y′−Elog⁡DGYXGXYx,y′,

(10) LdaGYX=−Elog⁡DGYXy,x′−Elog⁡DGXYGYXy,x′,

where *G*_*YX*_(*G*_*XY*_(*x*)) and *G*_*XY*_(*G*_*YX*_(*y*)) are the reconstruction of *x* and *y*, respectively.

### 3.2. Feature-Wise Loss

Deep feature matching in our work is inspired by the trick in [47] to improve the training of GAN, as well as the perceptual loss of [8, 14] for image style transfer. We perform the deep feature matching between the translated image and those randomly picked in the target domain, which are named unaligned data. We guide the generation with expected distribution, rather than the single exactly paired image. Let *y*_in_ and *y*_c_ be the input image and the conditioning image of the feature matching network *φ*. Respectively, *φ*_*l*_(*y*_in_) and *φ*_*l*_(*y*_c_) are the feature representation in layer *l* of network *φ*. *y*_in_ might be the real image or the fake image as defined in GAN [16] and *y*_c_ is the unaligned target of the input image *x* as defined afore or the aligned data when available. The feature discrepancy of layer *l* in the feature matching network *φ* is the Euclidean distance between the activation of *y*_in_ and *y*_c_:

(11) $$\begin{array}{c} l_{l}^{\varphi }\left(\;y_{in},y_{c}\;\right)=\frac{1}{C_{l}H_{l}W_{l}}{\left\|\;\varphi_{l}\left(\;y_{in}\;\right)-\varphi_{l}\left(\;y_{c}\;\right)\;\right\|}_{2}^{2}, \end{array}$$

where *C*_*l*_, *H*_*l*_, and *W*_*l*_ are filter amount, height, and weight, respectively. As the discriminator we proposed acts as a cross-domain transfer, features extracted by layers of this discriminator are both expressive and discriminative [48, 49]. Experiments shown in Section 5.3.1 on the configurations for feature comparator further validate this assumption; thus all feature mapping networks in our experiments share the same architecture with the discriminator network *D*. Then the total feature-wise loss is

(12) $$\begin{array}{c} L_{feat}=\underset{l}{\sum }w_{l}l_{l}^{D}, \end{array}$$

where the weighting parameters *w*_*l*_ describe the contribution of layer *l* to the sum of perceptual loss. In preliminary experiments we also try the style loss in [50] for comparison, only to find expensive computational cost and negligible improvement.

### 3.3. Pixel-Wise Loss

Although feature loss can better maintain higher frequency features, when employed alone it generally induces artifacts [44]. Thus mixing the feature loss with other losses is essential to guide the image generation. Empirical successes are obtained when pixel-wise loss *ℓ*_*k*_ is included [1, 13]. This is because the pixel loss could provide gradients that alleviate the unstable training problem of GANs to some extent:

(13) Lpix=lkx,GYXGXYx+lky,GXYGYXy.

When aligned data is available, the first term alone is sufficient to construct a supervised loss. Besides, we use *ℓ*1 distance as the pixel-wise loss in our paper for the purpose of encouraging less blur than *ℓ*2 loss.

### 3.4. Full Objective

As for the aligned image-to-image translation, we aim to optimize the following minimax objective:

(14) $$\begin{array}{c} \underset{G}{\min}\underset{D}{\max}\;L_{align}=\underset{G}{\min}\underset{D}{\max}\;L_{align}^{cGAN}+L_{pix}+L_{feat}. \end{array}$$

When it comes to the unaligned cases, full objective can be defined as

(15) LunalignG=LdaGXY+LdaGYX+Lpix+Lfeat,

(16) LunalignD=LdaDX+LdaDY,

In practice, both the discriminator *D* and the generator *G* are iteratively optimized by stochastic gradient decent (SGD) [47].

## 4. Implementation

### 4.1. Network Architectures

Our architecture consists of three kinds of components: discriminators, generators, and the feature comparators. As for the network architectures of discriminators and the generators, we build on the works of [18, 20], which had performed extensive studies on the discriminator and generator and shown compelling results on the aligned and unaligned image translation, respectively. Here we focus on the configuration of the feature comparators, and we find that the one shares the network architecture of the discriminator is sufficient to capture the deep representations while avoiding extra computations. We employ the instance normalization for generators in both aligned and unaligned image translations. Besides, 70 × 70 patch-level discriminator is adopted for fewer parameters. When it comes to the unaligned image-to-image translation, architectures above are coupled to construct a closed cycle, as shown in Figure 2.

### 4.2. Training Details

All of our networks are trained from scratch with weights initialized from a Gaussian distribution *N* (0, 0.02^2^). Alternate SGD [47] and Adam solver [51] with a momentum term of 0.5 and a learning rate of 2 × 10^−4^ are applied to *D* and *G*. Training epochs vary with the dataset size of different tasks. For our implementation we employ Tensorflow [52] and cuDNN [53].

## 5. Experiments

To assess our proposed approach, we apply it to diverse image-to-image translation tasks on both aligned and unaligned datasets and compare it with several baselines that achieve state-of-the-art performance in specific image translation tasks. First comparison concerning the configuration of feature comparator network is presented. Then ablation study is performed to empirically demonstrate the effectiveness of the ensemble loss function. To validate the universality of the proposed method, we experiment on the cityscapes dataset with aligned and unaligned setting, respectively. Finally, we present the quantitative comparisons with baselines on the image deraining or desnowing tasks. Most previous works on common framework for image translations traditionally require supervised datasets, while ours give comparable qualitative images even in the absence of aligned data. When having access to the ground-true images, superior results can be attained through our method.

### 5.1. Evaluation Metrics

To validate the proposed approach for both aligned and unaligned image-to-image translation, we adopt qualitative and quantitative measurements to evaluate the translated images. For image deraining and desnowing, we calculate the quantitative metrics such as Structural Similarity Index (SSIM) [54], Peak Signal to Noise Ratio (PSNR), Universal Quality Index (UQI) [55], and Visual Information Fidelity (VIF) [56] since ground-true is available. For qualitative experiments, visual assessment is used.

### 5.2. Baselines

*CNN*. The CNN we refer to here is the model that sets the adversarial loss and perceptual loss of our model to be zero; thus it is equivalent to a traditional CNN. 

*Pix2pix [18]*. Pix2pix is used as our baseline from two perspectives. First, when it comes to the unaligned image-to-image translation, we use pix2pix as our upper bound to demonstrate our performance since it is supervised and trained. Second, concerning the aligned setting, we compare our method, which is conditioned on the unaligned target and is domain-adversarially trained, with pix2pix that is conditioned on the input and trained with plain adversarial loss. 

*CE [1]*. Being similar to pix2pix, context-encoder employs the adversarial loss and pixel-wise loss together. It was specifically proposed to predict the missing regions of the corrupted images based on the surrounding context. 

*BiGAN [57]*. Like our model, BiGAN does not learn the mapping from noise to images but maps inversely. The difference between us is that we map the input image to the target image rather than the noise vector, from which we learn the mapping back to the input. 

*CycleGAN [20]*. Like the pix2pix [18], Zhu et al. use a combination of plain adversarial loss and pixel loss, whereas we consider a domain-based adversarial loss instead and an extra feature loss. 

*ID-CGAN [58]*. ID-CGAN is specifically proposed to solve the image deraining problem and has achieved the state-of-the-art performance. Like our method, this concurrent work employs the feature matching loss for deep image representations. By contrast, we choose simpler features than that of the ID-CGAN and condition the discriminator on the available images in the target domain. 

*PAN [59]*. It is another concurrent work that combines the adversarial loss and distances in deep feature representation for several image-to-image transformation tasks.

### 5.3. Comparison with Baselines

To assess the proposed approach, we compare it with some recent state-of-the-art methods on several specific image translation tasks, including the image inpainting, map ↔ aerial translation, semantic label ↔ image conversion, and image deraining or desnowing. For fair comparisons, we implement objectives of all the baselines with the same networks architecture and training details used in our model on each task unless otherwise specified.

#### 5.3.1. Image Inpainting

We perform the image inpainting with the aim of choosing the appropriate configuration for the feature comparator as well as validating the effectiveness of our method. Dataset used in this experiment is CelebA [60], which is a satisfying visual assessment tool as we humans are sensitive to flaws in face images.

**Figure 3:**
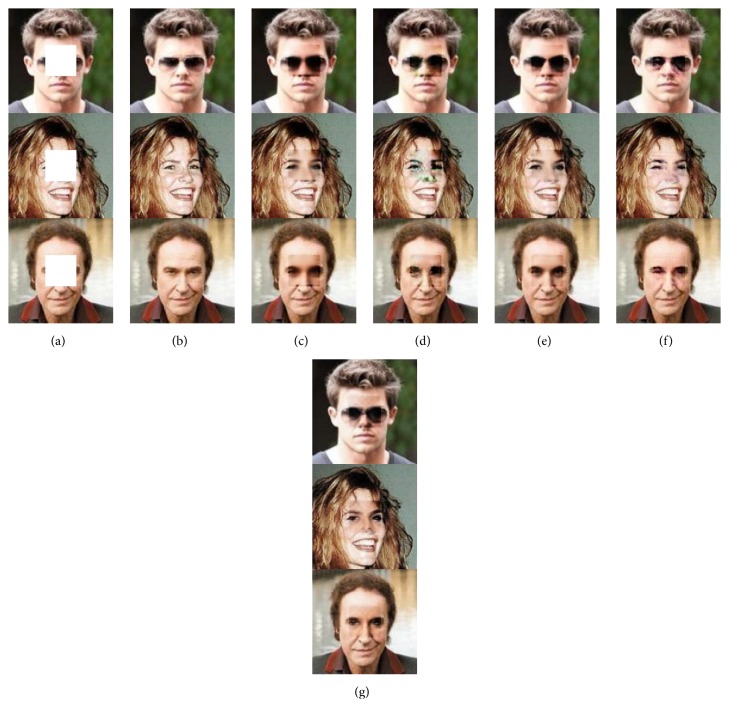
Different models and several configurations of our feature comparator for image inpainting: (a) input; (b) ground-true; (c) context-encoder [1]; (d) pix2pix [18]; (e) our model with all the ReLU layers of the discriminator; (f) our model with only the last ReLU layer of the discriminator; and (g) our model with all the last ReLU layer of each block of VGG19 [46].

As shown in Figure 3, CE [1] predicts blurred central regions as its reconstruction is implemented by *ℓ*_2_-based pixel loss, while pix2pix introduces unsatisfying artifacts due to heavy emphasis on adversarial loss. Then we proceed to choose appropriate configuration by comparing different networks or layers for feature representation discrepancy calculating. Figure 3 shows that merely using the activation of the last layer of the discriminator (Ours_ll) results in fine details insufficiency, while considering all the last activated layers of every block in the pretrained VGG19 [46] (Ours_vgg) tends to induce checkerboard artifacts and color distortion. Therefore, we choose the discriminator network *D* as the feature matching network in all our experiments.

#### 5.3.2. Map *↔* Aerial Photograph

**Figure 4:**
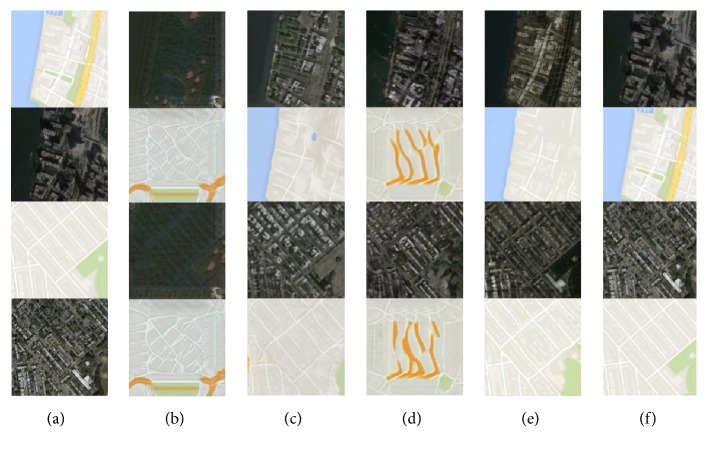
Qualitative performance on the map *↔* aerial photograph translation: (a) input; (b) unconditional GAN; (c) unconditional GAN with pixel loss (CycleGAN [20]); (d) GAN with conditioning on the input image; (e) GAN with conditioning on the unaligned target as well as the feature-wise loss (ours); and (f) supervised pix2pix.

We perform ablation study to empirically demonstrate that the ensemble loss function is a simple yet powerful approach to achieve substantial performance boost. Results are compared in the task of map *↔* aerial photograph translation on the dataset directly captured from Google Maps [18]. Though the datasets of maps and aerial photograph are aligned, we set the unaligned configuration to mimic the reality as supervised data are generally unavailable. As can been seen in Figure 4, unconditional configuration induces mode collapse but improves greatly when pixel-wise reconstruction is additionally employed, which is referred to as CycleGAN [20]. We conjecture that the cycle-consistent loss, which is made up of the reconstruction loss of bidirection, reduces the space of mapping from an individual input to the desired output. When conditional information is taken into consideration, realism increases. However, mode failure is inevitable in the other mapping direction unless cyclic loss is enforced. Impressive translated images, which are nearly matched with the ground-true, can be attained when we further augment the feature loss. In general, the conditional version is superior to its unconditional counterpart, and the gaps in between can be shrunk by conditioning the discriminator or introducing a pixel-wise error. Indeed, a complete model as ours is able to yield compelling results.

#### 5.3.3. Label *↔* Facades Photograph

The proposed method does not require aligned data in the target domain. At the same time, supervised data can be easily incorporated when they are available. On the task of label *↔* facades photograph conversion, we first experiment with the unsupervised setting and then switch to the supervised case to demonstrate the flexibility of our method.

**Figure 5:**
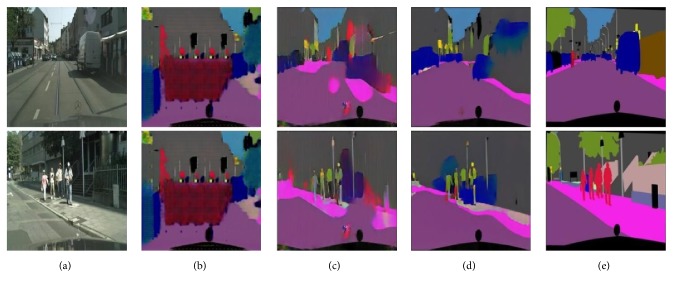
Qualitative examples on the aligned dataset without input-target supervision (except for pix2pix): (a) input; (b) BiGAN [57]; (c) CycleGAN [20]; (d) ours; and (e) ground-true.

When they refer to the unaligned setting on the supervised dataset, images in the target domain are shuffled to be unaligned with the input during training. The purpose is that the results of supervised learning, which is trained on the aligned data, could be used for an indicator to intuitively show the performance gap between ours and those of supervised learning. As can be seen in Figure 5, BiGAN [57] could barely produce the reasonable results as no bidirection closed loop constraint is enforced. Even though CycleGAN [20] is able to tackle this issue by cycle-consistent enforcement, the edges are blurry since the cyclic loss is pixel-based. Compared with our method, CycleGAN shows visual inferiority where ambiguous labels and discontinuous boundary are easily observed. On the whole, the proposed method is able to achieve more desirable translated images, which are much closer to those of the supervised pix2pix, yet without supervision.

**Figure 6:**
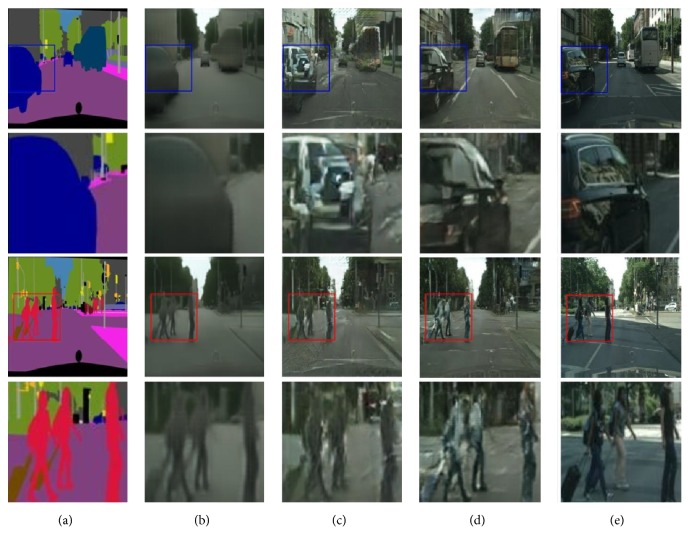
Different approaches to labels → cityscapes translation: (a) input; (b) CNN; (c) pix2pix; (d) ours; and (e) ground-true.

Figure 6 shows the comparison on the aligned data. While pix2pix [18] is able to produce much sharper results than those of CNN by enforcing a conditional adversarial loss, which trains the discriminator to reject obvious artifacts, translated images of pix2pix [18] include hallucinated objects. For better visual comparison, zoomed versions of certain regions-of-interest are accordingly shown below the results. On the whole, any of the baselines above is competitive with the proposed method.

#### 5.3.4. Image Deraining and Desnowing

**Figure 7:**
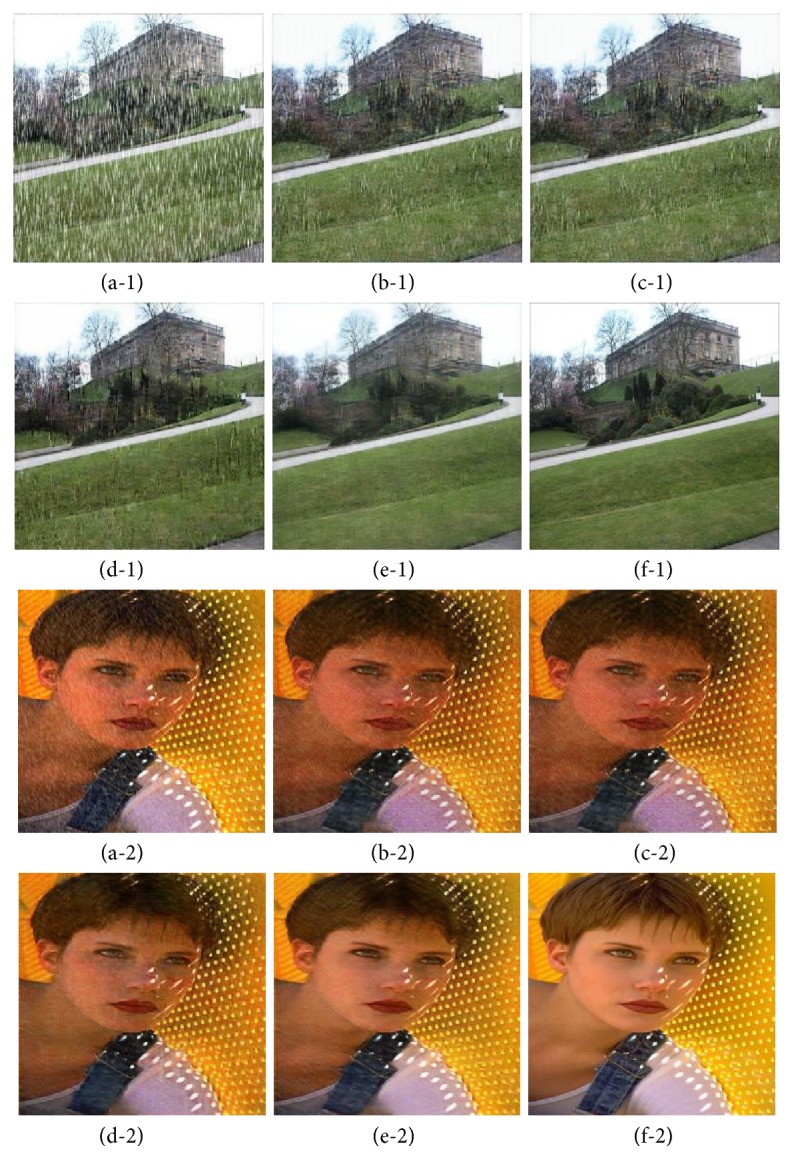
Example results of different models for image deraining (desnowing): (a) input; (b) CNN; (c) ID-CGAN [58]; (d) PAN [59]; (e) ours; and (f) ground-true.

We present quantitative comparison in this subsection. Datasets that include both synthetic and real data for image deraining or desnowing are from [58]. Metrics we choose for quantitative evaluations are PSRN, SSIM, UQI, and VIF as ground-trues are available and well aligned in pixel space on the synthetic dataset. Results are reported in Table 1, where the outperformance of our method against other baselines on all the metrics can be clearly observed. Visual comparisons are shown in Figure 7, making the contrast even more distinct. CNN and ID-cGAN [58] result in relatively poor performance as the raindrops or snowflakes are still clearly observed. Even though PAN [59] is able to reduce the intensity of raindrops and snowflakes, it tends to result in artifacts. In comparison, our method can successfully remove most of the noise while retaining the background details of the input images.

## 6. Conclusions and Future Work

We develop a cGAN-based framework that is applicable to both aligned and unaligned image-to-image translation with domain-adversarial training. Compelling results suggest that neither the feature representations nor the image priors require hand-engineering in our framework. The former can be captured by discriminator that is trained for domain adapting and detecting generated images, while the latter can be learned by conditioning the discriminator on unaligned data from the target domain. Experiments demonstrated that our method can generate realistic images with desired style on diverse image translation tasks without supervised data or manual intervention. Despite being suitable for learning global style, our method, like other cycle-consistent models, is weak at specific attribute editing which might be addressed by representations disentangling and multimodal generation.

## Figures and Tables

**Table 1:** Quantitative results of different approaches, evaluated on image deraining (desnowing).

	CNN	ID-CGAN [58]	PAN [59]	Ours
PSNR	22.98	22.73	23.35	**24.34**
SSIM	0.74	0.81	0.83	**0.89**
UQI	0.51	0.64	**0.66**	**0.66**
VIF	0.35	0.41	0.41	**0.51**
